# Oxygen Isotopic Composition of U_3_O_8_ Synthesized From U Metal, Uranyl Nitrate Hydrate, and UO_3_ as a Signature for Nuclear Forensics

**DOI:** 10.1021/acsomega.1c07042

**Published:** 2022-02-22

**Authors:** Maor Assulin, Ruth Yam, Eyal Elish, Aldo Shemesh

**Affiliations:** † Department of Earth and Planetary Sciences, Weizmann Institute of Science, 234 Herzl Street, P.O. Box 26, Rehovot 7610001, Israel; ‡ Analytical Chemistry Department, Nuclear Research Center Negev (NRCN), P.O. Box 9001, Beer-Sheva 84190, Israel

## Abstract

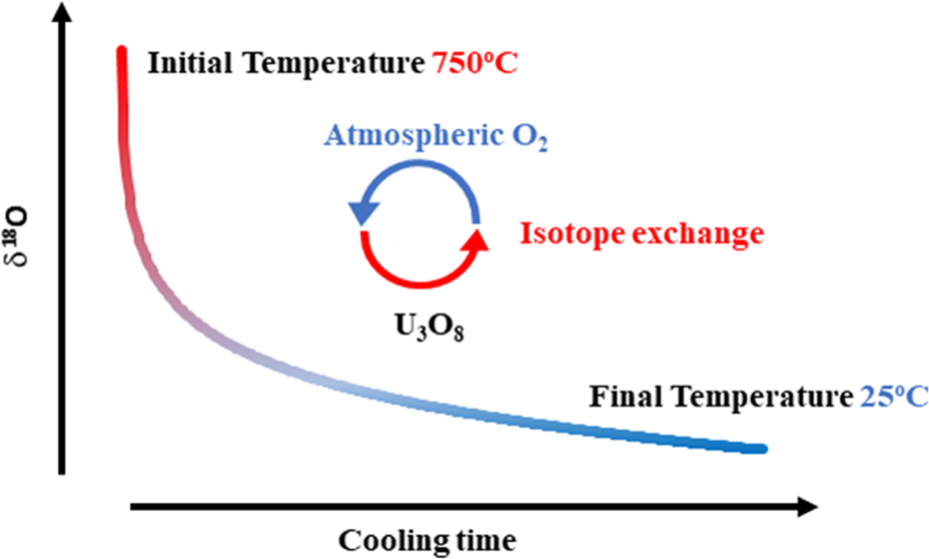

Triuranium octoxide (U_3_O_8_) is one of the main compounds in the nuclear fuel cycle. As such, identifying its processing parameters that control the oxygen isotopic composition could be developed as a new signature for nuclear forensic investigation. This study investigated the effect of different synthesis conditions such as calcination time, temperature, and cooling rates on the final δ^18^O values of U_3_O_8_, produced from uranium metal, uranyl nitrate hydrate, and uranium trioxide as starting materials. The results showed that δ^18^O of U_3_O_8_ is independent of the above-listed starting materials. δ^18^O values of 10 synthetic U_3_O_8_ were similar (9.35 ± 0.46‰) and did not change as a function of calcination time or calcination temperature. We showed that the cooling rate of U_3_O_8_ at the end of the synthesis process determines the final oxygen isotope composition, yielding a significant isotope effect on the order of 30‰. Experiments with two isotopically spiked 10 M HNO_3_, with a difference of δ^18^O ∼75‰, show that no memory of the starting solution oxygen isotope signature is expressed in the final U_3_O_8_ product. We suggest that the interaction with atmospheric oxygen is the main process parameter that controls the δ^18^O value in U_3_O_8_. The uranium mass effect, the tendency of uranium ions to preferentially incorporate ^16^O, is expressed during the solid–gas oxygen exchange, which occurs throughout cooling of the system.

## 1 Introduction

Nuclear forensics is essential for investigating nuclear material found outside of regulatory control or used in an act of terrorism. Tens of illicit trafficking incidents related to natural, depleted, or enriched uranium have been reported since 2019.^1^ In the last three decades, nuclear forensic signatures such as elemental, isotopic, and trace element compositions of various uranium matrices were found to be valuable to understand the material history. While the rare earth element pattern and strontium and neodymium isotope ratios are unique signatures related to geographic location,^2−5^ oxygen and lead are related to both geographic location and production processes.^6−17^ The isotopic ratio of uranium/thorium is used to determine the material age,^18^ and the elemental composition of the impurities provides information regarding the production process.^18−23^

The oxygen isotopic composition is affected by various chemical and physical reactions (e.g., isotope exchange and kinetic effects), leading to preferential isotope distribution between the chemical reagents and final uranium oxide phases, and thus, it can be utilized as an additional signature for the processes involved.

The production processes of uranium fuel pellets (UO_2_) consist of several stages, starting with milled uranium ore to produce intermediate products, mainly uranyl nitrate hydrate (UNH), ammonium diuranate (ADU), and uranium peroxide.^24−26^ Uranium oxides such as UO_3_, U_3_O_8_, and UO_2_, in the form of powders, are intermediate compounds within uranium ore processing and nuclear fuel production cycle, regardless of the above-mentioned starting materials.^24−26^ U_3_O_8_ is almost ever present in the production cycle of nuclear fuel. Furthermore, U_3_O_8_ is a major compound in nuclear waste management due to its thermal and chemical stability.^24−28^ All production processes of U_3_O_8_ compounds in the nuclear industry involve three major parameters: calcination temperature, calcination time, and the cooling rate of the products.^26^

Dierick et al.^12^ synthesized U_3_O_8_ samples from ADU and uranium peroxide at 750 °C for 3 h, under atmospheric conditions. The isotopic composition of the U_3_O_8_ product exhibited a wide range of δ^18^O values, from −22.45 to 2.45‰ (vs Vienna Standard Mean Ocean Water (VSMOW)), independent either of the starting materials or the isotope composition of the solutions used. Plaue et al.^11^ synthesized U_3_O_8_ from UO_2_ and measured δ^18^O values of 18.4 and 20.0‰ at 700 and 800 °C under dry air, after 90 and 96 h, respectively. Klosterman et al.^16^ synthesized U_3_O_8_ from metastudtite (UO_2_(O_2_)·2H_2_O) at a temperature range of 600–800 °C, up to 100 h under dry air, yielding lower δ^18^O values, in the range of 14.7–17.9‰. The retrograde isotope effect was invoked so as to explain the difference between the above studies.^16^ The wide range of δ^18^O values measured for the synthesized U_3_O_8_ samples under a relatively narrow range of temperatures is challenging if the links between the process and the materials involved have to be established.

Indications for fast oxygen exchange between gaseous O_2_ and solid U_3_O_8_ have been reported,^30,31^ where U_3_O_8_ was synthesized from amorphous-UO_3_ for 30 h at 600 °C and 5 h at 700 °C, under vacuum. As a function of temperature, the half-exchange times of labeled ^18^O with the synthesized U_3_O_8_ were effectively exponential at all degrees of exchange. The exchange was higher than 94% in less than 30 min at 525 °C. It was concluded that U_3_O_8_ contains several types of oxygen in the lattice; however, all react equivalently to exchange and have very similar binding energies.

This study focuses on the isotope signature change resulting from the manufacturing processes of U_3_O_8_ under different temperatures, calcination times, cooling rates, and initial solutions. We synthesized U_3_O_8_ originating from various UNH and UO_3_, via several processes commonly used in the uranium nuclear fuel cycle. We dissolved pure U metal in isotopically spiked HNO_3_ to affect the uranyl ion and assess the incorporation of oxygen isotopes into U_3_O_8_.

## 2 Materials and Methods

U_3_O_8_ was synthesized from uranyl nitrate hydrate and UO_3_. The UNH samples were prepared by bringing to dryness the 10 g/L uranium standard solutions (SPEX, CertiPrep, Fisher Scientific). Pure U metal (Merck, Germany) was dissolved in 10 M nitric acid (Honeywell Fluka, Fisher Scientific), which was isotopically spiked to have δ^18^O water compositions of −50 and +25‰, for the synthesis of U_3_O_8_ via UNH. All of the samples were stored in a vacuum desiccator.

### 2.1 Preparation of Uranyl Nitrate Hydrate

Eight different uranyl nitrate hydrate samples were prepared by evaporating uranium single element standard solution (SPEX) in a Pyrex beaker dipped in a sand bath, placed on a hot plate, applying a temperature range of 40–85 °C until complete dryness. In addition, two spiked nitric acid solutions, with known δ^18^O values, were used for the dissolution of uranium metal. The preparation conditions and the final chemical composition of the UNH are presented in Table 1. The chemical structure of the products was determined by X-ray diffraction (XRD) measurements (Rigaku, Ultima III, 40 kV/40 mA).

**Table 1:** Synthesis Conditions and Chemical Composition of UNH

sample	temperature (°C)	drying duration (h)	chemical formula
ML-1	80	168	100% UO_2_(NO_3_)_2_·6H_2_O
MH-1	80	168	100% UO_2_(NO_3_)_2_·6H_2_O
SPEX-40	85	54	17.6% UO_2_(NO_3_)_2_·2H_2_O
			66.0% UO_2_(OH)_2_
			16.5% UO_2_(NO_3_)_2_·6H_2_O
SPEX-50	80	30	92.6% UO_2_(NO_3_)_2_·3H_2_O
			7.4% UO_2_(OH)_2_
SPEX-60	80	30	90.2% UO_2_(NO_3_)_2_·3H_2_O
			9.8% UO_2_(OH)_2_
SPEX-70	80	30	59.6% UO_2_(NO_3_)_2_·3H_2_O
			23.9% UO_2_(NO_3_)_2_·6H_2_O
			16.6% UO_2_(OH)_2_
SPEX-80	80	30	75.6% UO_2_(NO_3_)_2_·3H_2_O
			21.0% UO_2_(NO_3_)_2_·6H_2_O
			3.3% UO_2_(OH)_2_
UNH-S-T-40	40	120	100% UO_2_(NO_3_)_2_·3H_2_O

MH-1 and ML-1 were prepared by dissolving U metal in isotopically heavy (HW) and light (LW) ^18^O-labeled 10 M nitric acid solutions. The solutions were prepared from 70% (15.8 M) analytical-grade nitric acid diluted to 10 M nitric acid using nano pure water (NPW); 15.8 M nitric acid was spiked with water possessing δ^18^O of −140 and 5515‰. Hence, each spiked solution contained water from three different sources: original 15.8 M water, dilution water (NPW), and enriched or depleted spiking water solutions. The labeled solutions were equilibrated for 107 days at 25 °C. Isotope equilibrium in H_2_O–HNO_3_ δ^18^O is usually achieved after ∼100 h and the fractionation (ε) of HNO_3_–H_2_O is 22.5‰ at 25 °C.^32−34^ Based on 10 measurements, δ^18^O of the nitrate group of commercial acids is, in general, in the range of 20.0–28.0‰,^32^ which allows calculating the δ^18^O of the original acid water to be ∼1 to 5‰, using the NPW with a δ^18^O value of 0.5‰. The expected δ^18^O values of the final acid, at equilibrium, in the HW solution, are 25 and −50‰ in the LW solution. The expected δ^18^O values of the nitrate group in the HW solution are 47.5 and −27.5‰ in the LW solution. MH-1 was prepared by dissolving 325 mg of U metal in 32.5 mL of HW, while ML-1 was prepared by dissolving 325 mg of U metal in 32.5 mL of LW at room temperature, to match the concentration of 10 g/L. These two solutions were used to synthesize UNH, as is detailed in Table 1.

### 2.2 Preparation of UO_3_

Six samples of uranium trioxide (UO_3_) were prepared from UNH at 400–450 °C, producing an amorphous phase. About 150 mg of UNH was oxidized under atmospheric conditions in a Pt crucible in a preheated furnace for 4 h.

### 2.3 Preparation of U_3_O_8_

Twenty-nine samples of triuranium octoxide (U_3_O_8_) were synthesized using two pathways: UNH and UO_3_. U_3_O_8_ originating from UNH was prepared at different calcination temperatures, calcination times, and cooling rates. About 150 mg of UNH were placed in a Pt crucible in the furnace preheated to 650–850 °C for 0.5–168 h. Several cooling rates, from 750 to 25 °C, were applied. The synthesis conditions are detailed in Table 2.

**Table 2:** Starting Material and Conditions That Were Used to Synthesize U_3_O_8_

U_3_O_8_ sample	starting material	calcination temperature (°C)	calcination time (h)	cooling time from 750 to 25 °C (min)
SPEX-52	SPEX-50	750	4	7
SPEX-62	SPEX-60	750	4	7
SPEX-72	SPEX-70	750	4	7
SPEX-82	SPEX-80	750	4	7
T-1	T-8-UO_3_	750	4	7
T-3	T-11-UO_3_	750	4	7
T-4	T-12-UO_3_	750	4	7
T-5	T-7-UO_3_	750	4	7
T-6	T-10-UO_3_	750	4	7
C-T-1	UNH-S-T-40	650	2	7
C-T-2	UNH-S-T-40	700	2	7
C-T-3	UNH-S-T-40	750	2	7
C-T-4	UNH-S-T-40	800	2	7
C-T-5	UNH-S-T-40	850	2	7
D-T-1	UNH-S-T-40	750	0.5	7
D-T-2	UNH-S-T-40	750	1	7
D-T-3	UNH-S-T-40	750	2	7
D-T-4	UNH-S-T-40	750	4	7
D-T-5	UNH-S-T-40	750	6	7
SPEX-42	SPEX-40	750	4	7
SPEX-43	SPEX-41	750	4	7
ML-3	ML-1	750	4	7
MH-3	MH-1	750	4	7
Long D-T-3	D-T-3	750	168	7
U_3_O_8_-I	UNH-S-T-40	750	4	2027
U_3_O_8_-II	UNH-S-T-40	750	4	247
U_3_O_8_-III	UNH-S-T-40	750	4	256
U_3_O_8_-VI	UNH-S-T-40	750	4	454
U_3_O_8_-IV	UNH-S-T-40	750	4	2.5

X-ray diffraction (XRD) was applied to determine the structural phase of the uranium oxides. XRD analyses (Rigaku, Ultima III) were conducted on samples weighing several milligrams under an atmosphere environment by continuous scanning at 40 kV/40 mA in the range of 10–80° at a rate of 2°/min.

### 2.4 Oxygen Isotope Measurement

Oxygen analysis of uranium oxides was performed with an isotope ratio gas chromatography mass spectrometer (irmGCMS, Thermo Scientific Delta Plus Advantage) and an IR CO_2_ laser (10.6 μm New Wave Research—25 W). The method has been previously described in detail.^15,35,36^ The synthesized U_3_O_8_ samples are fine-grained and no additional treatment was needed prior to the LF-IRMS analysis. U_3_O_8_ samples (1000–1700 μg) and SiO_2_ samples (200–560 μg) were placed in Nickle holders in a stainless steel chamber and heated at 80 °C overnight under high vacuum. Prefluorination was performed three times for the entire cell, with BrF_5_. The CO_2_ laser provides heating for a single sample reaction under a 90 Torr BrF_5_ atmosphere. The released oxygen was purified by liquid nitrogen traps, concentrated on a molecular sieve cooled in liquid nitrogen, and transferred to a mass spectrometer through a gas chromatograph column for isotope measurement, in a continuous flow mode. The international SiO_2_ standard NBS-28 (δ^18^O = 9.58‰)^37^ was introduced in each batch, for both consistency and calibration. The measured values were expressed in δ-notation in permil relative to Vienna Standard Mean Ocean Water (VSMOW). The long-term standard deviation (SD) for NBS-28 was 0.36‰. Each U_3_O_8_ sample was run at least in triplicate and the SD is reported for each sample.

## 3 Results and Discussion

### 3.1 δ^18^O and XRD of U_3_O_8_ from UNH and UO_3_

XRD diffractograms of UNH samples indicated uranyl nitrate structures containing 2–6 water molecules, uranyl hydroxide, or a mixture of various proportions (Table 1). UO_3_ samples exhibit different degrees of crystallinity, dominated by the amorphous phase. On the other hand, all of the resulting U_3_O_8_ samples were identical, consisting of a single-phase α-U_3_O_8_, irrespective of the UNH or UO_3_ phase from which it had been prepared. The range of temperatures in which U_3_O_8_ samples were synthesized by both routes, UNH or UO_3_, ensured a complete conversion of the starting materials into a single phase of U_3_O_8_.^27^ This result is in good agreement with a previous study^29^ that examined the morphological changes in α-U_3_O_8_ synthesized from amorphous-UO_3_, under four calcination temperatures 650, 700, 750, and 800 °C under purified air. It showed similar XRD spectra in this range of temperatures and that the UO_3_ samples were fully converted to α-U_3_O_8_ above 600 °C and did not change up to 800 °C. The δ^18^O values of the U_3_O_8_ samples are presented in Table 3 (starting from UNH) and in Table 4 (starting from UO_3_). The average δ^18^O value of all six U_3_O_8_ samples prepared from UNH is 8.15‰ with a standard deviation of 0.66‰ (*n* = 21). The oxygen yields were identical to those of NBS-28. This result suggests that all of the oxygen was liberated from the analyzed samples and that the reaction was complete. Regarding sample size, the amount of liberated oxygen was linear, ranging from 1000 to 1700 μg of U_3_O_8_, indicating similar stoichiometry of U_3_O_8_ prepared from different UNH.

**Table 3:** δ^18^O (in ‰ Relative to VSMOW) Values for U_3_O_8_ Samples Synthesized from UNH

sample	δ^18^O (‰ VSMOW)	SD (‰)	# of replicates
SPEX-52	8.66	0.62	5
SPEX-62	8.01	0.70	3
SPEX-72	8.00	0.77	4
SPEX-82	8.22	0.63	5
SPEX-42	7.70	0.42	4
aNBS-28	9.54	0.36	22

a NBS-28 has an assigned isotope value of 9.58 ± 0.09‰ as an international standard.^36^

**Table 4:** δ^18^O (in ‰ Relative to VSMOW) Values for U_3_O_8_ Samples Synthesized from UO_3_

sample	δ^18^O (‰ VSMOW) of the starting material (UO_3_)	δ^18^O (‰ VSMOW) of the final material (U_3_O_8_)	SD (‰)	# of replicates (U_3_O_8_)
T-1	4.1	9.10	0.48	3
T-3	8.9	8.87	0.47	4
T-4	13.4	9.40	0.14	4
T-5	16.1	9.35	0.36	3
T-6	24.9	8.31	0.23	3
SPEX-43	–9.6	8.57	0.30	4
NBS-28		9.49	0.36	16

The average δ^18^O of U_3_O_8_ samples prepared from UO_3_ was found to be 8.94 ± 0.50‰ (*n* = 21), despite a wide range of δ^18^O values, from 24.9 to −9.6‰, of the starting UO_3_ materials (Table 4).

**Figure 1:**
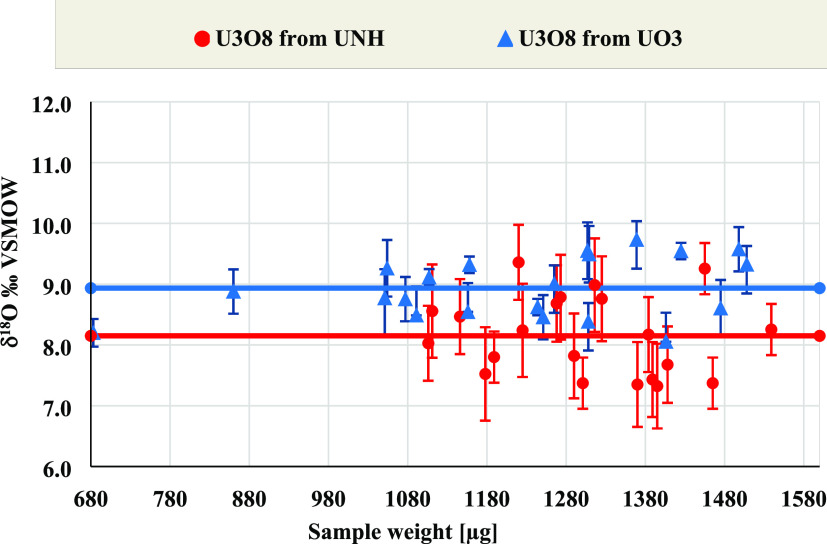
δ^18^O vs the expected oxygen content, calculated by the weight of U_3_O_8_ synthesized from UNH and UO_3_.

The average δ^18^O values of U_3_O_8_ synthesized from UO_3_ and UNH are 8.94 ± 0.50‰ and 8.15 ± 0.66‰, respectively (Figure 1). The similarity between the δ^18^O values of U_3_O_8_ obtained by both preparation routes suggests that the final δ^18^O value is independent of the starting material. It is also independent of the original δ^18^O value of UO_3_ from which it was prepared. The results point out to atmospheric oxygen as a common external source that determines the final oxygen isotopic composition.

### 3.2 Dissolution of Uranium Metal in Spiked HNO_3_

The similarity in the δ^18^O values obtained for both preparation routes (UNH and UO_3_) suggests that the oxygen isotopic composition of U_3_O_8_ is independent of the starting material δ^18^O value. Thus, a controlled experiment of dissolving a pure uranium metal in two isotopically different nitric acids was conducted to impose an isotope signature on the uranyl ion and follow it through the conversion to U_3_O_8_.

**Figure 2:**
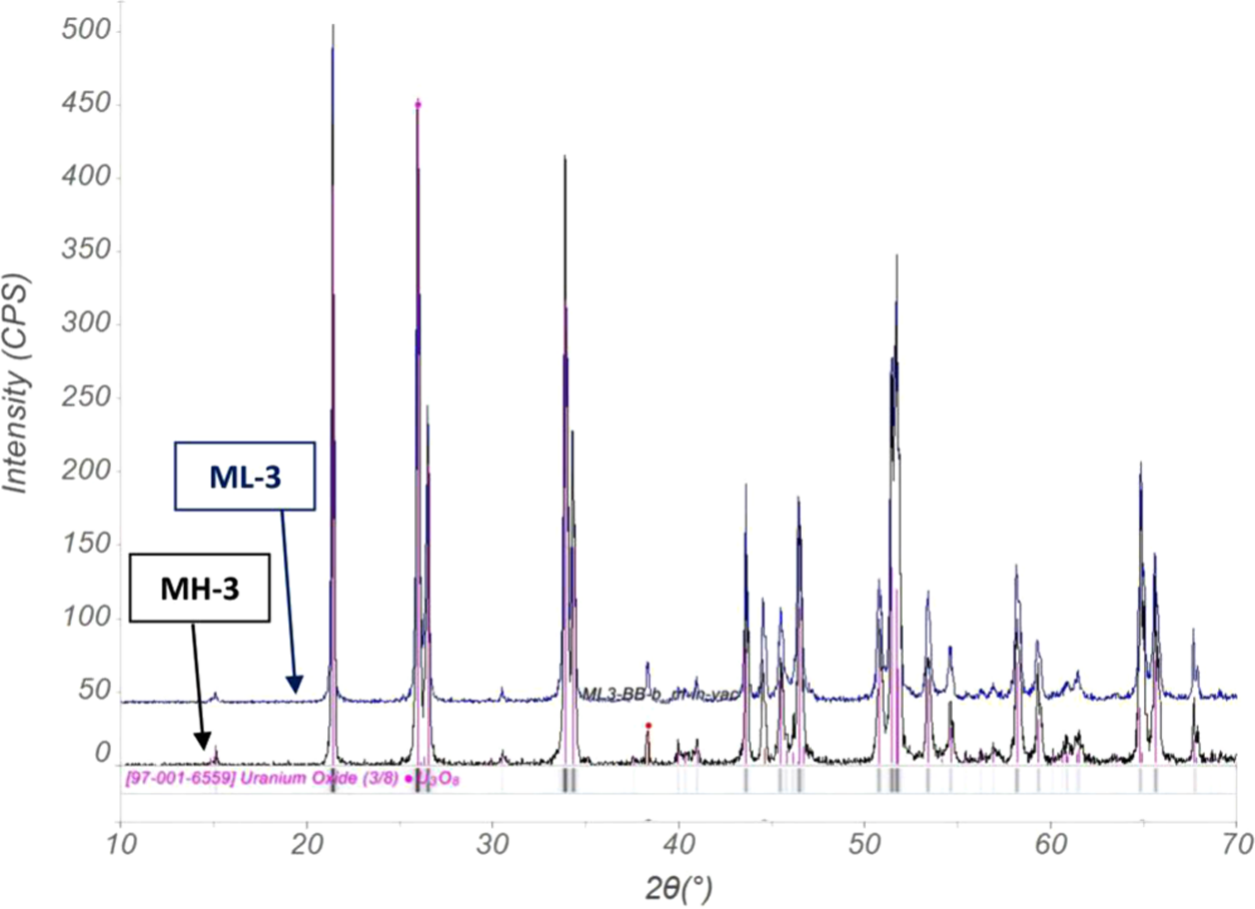
XRD analysis of U_3_O_8_ samples prepared from heavy water (MH-3) and light water (ML-3).

Two UNH samples were prepared by dissolving uranium metal in spiked 10 M HNO_3_, with H_2_O having δ^18^O of +25.0 and −50.0‰ the oxygen isotopes of the nitrate groups were +47.5 and −27.5‰, as described in Section 2.1. The production of U_3_O_8_ from these two isotopically spiked solutions aimed to further test the hypothesis regarding the dependence of the δ^18^O value in U_3_O_8_ on the starting material. XRD patterns of the samples prepared from heavy water (MH-3) and from light water (ML-3; Figure 2) show identical crystallographic structure, α-U_3_O_8_, with similar spectra to those obtained for U_3_O_8_, synthesized from nonspiked solutions.

The δ^18^O values for MH-3 and ML-3 are indistinguishable, 10.09 ± 0.15‰ (*n* = 5) and 10.05 ± 0.50‰ (*n* = 4), respectively, even though the starting solutions were 75.0‰ apart. The oxygen yields after fluorination for both MH-3 and ML-3 were similar to those obtained for all synthesized U_3_O_8_ samples reported in this work. The spiked H_2_O in the solutions also impaired the isotope change on the HNO_3_ oxygens. The nitric acid was equilibrated for 4.5 months with the spiked water, much longer than needed for equilibration in the H_2_O–HNO_3_ system, as reported in ref (33). However, our results show no memory from the initial oxygen of the spiked acids (Table 3). We note that the final δ^18^O values of both U_3_O_8_ (MH-3 and ML-3) remain close to the δ^18^O of U_3_O_8_, which were synthesized by the route of UNH and UO_3_. The dissolution of uranium metal in the spiked solution implies that the final isotopic composition of U_3_O_8_ remains independent of the starting material since the oxygen sources of the uranyl ion were isotopically spiked with either H_2_O or HNO_3_. The results further stress the involvement of another source of oxygen during calcination, presumably atmospheric O_2_.

### 3.3 Effect of Temperature and Calcination Time on δ^18^O in U_3_O_8_

Equilibrium isotope fractionation factors and rates of isotopic exchange are fundamental for the interpretation of stable isotope data. Thus, we conducted a set of experiments under controlled calcination times and temperatures so as to understand the mechanism affecting the δ^18^O values of a single phase of α-U_3_O_8_. The kinetic study was designed to represent the range of time and temperature relevant to the nuclear industry.

**Figure 3:**
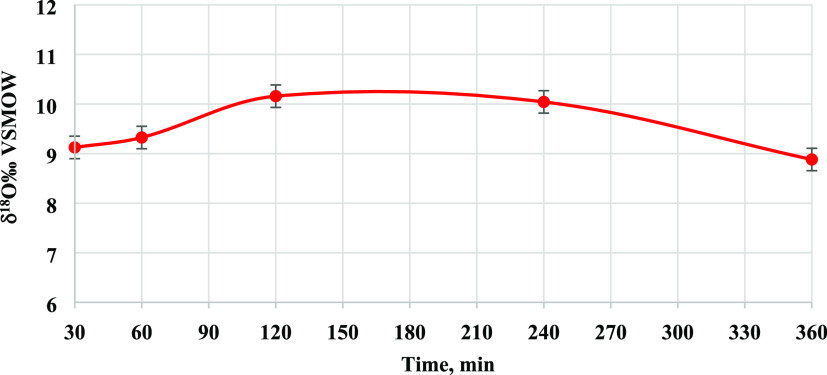
δ^18^O of U_3_O_8_ synthesized at 750 °C at different calcination times.

The δ^18^O values of the synthesized α-U_3_O_8_ at different calcination times are presented in Table 5 and Figure 3. The results show that δ^18^O varies by less than 1.5‰ throughout the duration of calcination, 30–360 min at 750 °C. We consider it as an insignificant minor change, relatively to the measured standard deviation (±0.34‰). The average δ^18^O of all samples is 9.50 ± 0.56‰.

**Table 5:** δ^18^O (in ‰ Relative to VSMOW) Values for α-U_3_O_8_ Synthesized at Different Calcination Temperatures and Times

sample				
calcination time: 2 h	temp. (°C)	δ^18^O (‰ VSMOW)	SD (‰)	# of replicates
C-T-1	650	8.30	0.74	3
C-T-2	700	8.37	0.81	7
C-T-3	750	9.02	0.45	9
C-T-4	800	10.67	0.42	4
C-T-5	850	9.66	0.53	5

calcination temp.: 750 °C	time (min)	δ^18^O (‰ VSMOW)	SD (‰)	# of replicates
D-T-1	30	9.12	0.55	4
D-T-2	60	9.32	0.11	3
D-T-3	120	10.16	0.51	3
D-T-4	240	10.04	0.41	3
D-T-5	360	8.88	0.12	3
long D-T-3	168 h	9.99	0.49	5

**Figure 4:**
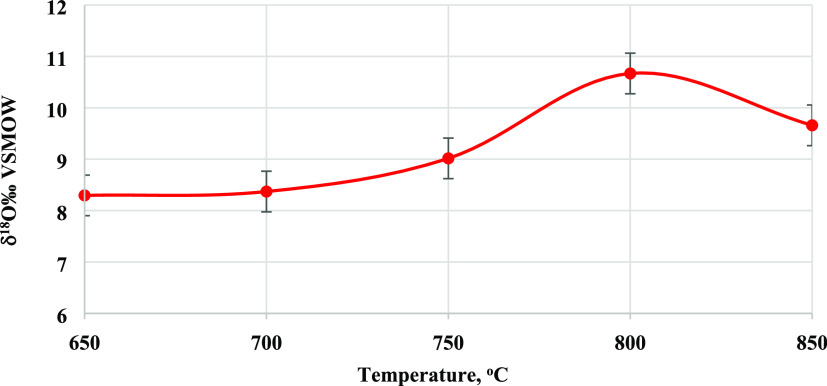
δ^18^O of U_3_O_8_ synthesized at 2 h at different calcination temperatures.

The δ^18^O values of α-U_3_O_8_ synthesized in different temperatures, between 650 and 850 °C for 2 h, are presented in Figure 4 and Table 4. The graph shows variability (within 0.5‰ error) between 600 and 750 °C, averaging 8.56 ± 0.34‰. The two samples prepared at temperatures above 750 °C show higher δ^18^O values, 10.67‰ at 800 °C and 9.66‰ at 850 °C.

A stable δ^18^O value is achieved within the first 30 min and remains constant over calcination periods of up to 6 h and calcination temperatures between 650 and 750 °C. These results point out a fast isotope exchange between solid and atmospheric O_2_. This conclusion is consistent with the fast oxygen exchange of U_3_O_8_ prepared from amorphous-UO_3_ under vacuum, reaching more than 94% exchange in about 23 min at 525 °C, reported by Lavut et al.^30^ This group also concluded that U_3_O_8_ contains several types of oxygen in the lattice; however, they all exchange equivalently and have similar binding energies. A higher degree of exchange can be assumed for our experiments, as it was conducted at higher temperatures. Our data are also in agreement with Plaue et al.,^11^ who suggested that oxygen isotope equilibrium of U_3_O_8_ with dry air was achieved in 6 h at 800 °C, and a similar apparent equilibrium was measured by Klosterman et al.,^16^ showing oxygen isotope compositions of U_3_O_8_ calcined at 700 °C between 30 min and 100 h in dry air.

The two U_3_O_8_, prepared at 800 and 850 °C, have δ^18^O values of 10.67 and 9.66‰, respectively, which are higher than the δ^18^O values of the samples prepared at lower temperatures (Table 5 and Figure 4). A similar ^18^O enrichment was reported by Klosterman et al.^16^ for the difference between samples prepared at 600–700 and 800 °C. We attribute this change to the preferential loss of the lighter oxygen isotope from the U_3_O_8_ lattice, which starts above 750 °C,^27^ and to the change in cooling rates, as discussed in Section 3.4.

We tested the stability of the isotopic signal of U_3_O_8_ for a longer period of calcination time, up to 168 h. Sample D-T-3, with an initial δ^18^O value of 10.16 ± 0.5‰, was calcined for 168 h and retained a δ^18^O value of 9.99 ± 0.49‰. Hence, the long-term stability of the exchange reaction can be extended to much longer periods.

### 3.4 Effect of the Cooling Rate of the Sample on δ^18^O Values in U_3_O_8_

The potential of fast isotope exchange during cooling as a significant factor controlling the final isotope value of U_3_O_8_ is evident, due to: (1) the fast isotope exchange process between U_3_O_8_ and atmospheric O_2_, (2) the lack of correspondence with the isotope values of the starting materials, (3) the fact that all isotope data from different preparation routes converge around the same isotope value, and (4) the 2‰ enrichment of samples prepared at high temperatures.

**Figure 5:**
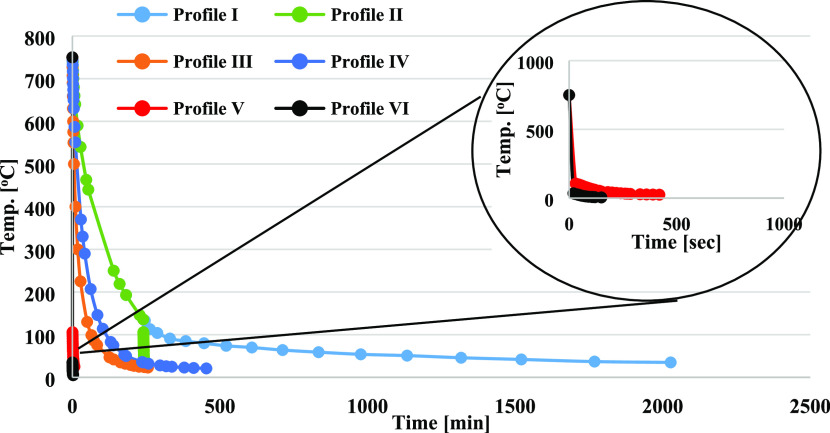
Cooling profiles applied to synthesized U_3_O_8_.

Several cooling rates, from 750 °C to room temperature, were tested to determine the relationship between the cooling rate and the final δ^18^O value of U_3_O_8_ (Table 6). The routine practice was to cool samples by removing them from the furnace at the preparation temperature, cooling to room temperature over 7 min, and storing them in a desiccator under vacuum. Thus, most of the results reported here (21 samples) correspond to this cooling profile. This cooling profile yielded an average δ^18^O value of 8.15 ± 0.66‰ when the preparation temperature was set to 750 °C. A faster cooling time of 2.5 min, followed by immediate transfer to an ice bath, produced a 4‰ heavier U_3_O_8_. On the other hand, the longest cooling period of 33 h to room temperature in an oven under an atmospheric environment yielded U_3_O_8_ with a depleted δ^18^O value of −22.22‰ (Table 6 and Figure 5). Applying cooling time in between the two extremes produced isotope values ranging from −22.2 to 12.3‰. Our results show that the cooling rate of U_3_O_8_ can change the δ^18^O value by ∼30‰, suggesting that the isotopic quenching over the cooling process is the main factor governing the final δ^18^O value in the production of U_3_O_8_. The rapid and continuous exchange with atmospheric oxygen during cooling, to a yet unknown closure temperature, produces a wide range of isotope values. As such, it may explain the discordant isotopic values published by Plaue et al.,^11^ Dierick et al.,^12^ and Klosterman et al.^16^ for α-U_3_O_8_ prepared under comparable conditions but probably under different, unreported, cooling rates.

**Table 6:** δ^18^O (in ‰ Relative to VSMOW) Values for α-U_3_O_8_ Synthesized at Different Cooling Profiles

sample	cooling profile	cooling time (min)	δ^18^O (‰ VSMOW)	SD (‰)	# of replicates
U_3_O_8_-I	profile I—cooling the furnace from 750 °C to room temperature with a closed door.	2027	–22.22	0.34	3
U_3_O_8_-II	profile II—cooling the furnace from 750 to 100 °C with a closed door and then to room temperature in the crucible outside the furnace.	247	–20.48	0.52	11
U_3_O_8_-III	profile III—cooling the furnace from 750 °C to room temperature with a partially open door (1 cm).	256	–16.24		1
U_3_O_8_-IV	profile IV—cooling the furnace from 750 °C to room temperature with a partially open door (1 cm).	454	–14.56	0.01	2
U_3_O_8_-V	profile V—removing the samples from the furnace at 750 °C and letting the samples cool to room temperature.	7	8.15	0.66	21
U_3_O_8_-VI	profile VI—removing the samples from the furnace at 750 °C and inserting the samples into an ice bath.	2.5	12.33	0.39	2

Currently, it has been possible to postulate a complete isotope exchange with atmospheric O_2_ (23.5‰) at 750 °C, based on the fast rate of this exchange.^37^ The resetting of the oxygen isotope toward lighter isotope compositions is supported by the study of ref (39). Their calculation based on the reduced partition function ratio for uranium oxides and water shows that uraninite is depleted in ^18^O with respect to the associated fluids at almost all temperature ranges (0–900 °C). As an example, exceptionally low δ^18^O values in natural uraninite, −20 to −30‰, were reported.^39^

Our results clearly demonstrate such a consistent “mass effect”,^38−41^ when we allow the produced U_3_O_8_ to cool slowly and perhaps reach an equilibrium with atmospheric O_2_ at low temperatures. Oxygen fractionation in the solids depends primarily on the vibrational frequencies of the bonds with uranium in the crystal. Kinetic processes such as the diffusion of O_2_ during the exchange largely depend on the solid particle size and organization, govern the rate, and point to a complex mechanism that is expressed in the final isotopic value. Such intense kinetic effects during cooling undermine the possibility of providing direct forensic geolocation information. However, our study highlights additional factors that control the fabrication process and expands the possible identification and characterization of nuclear production plants. Practically, this additional factor, the cooling time at the end of the production stage of U_3_O_8_, can be collected from different nuclear production plants and added to a worldwide nuclear forensics database.

## 4 Conclusions

This study examined the effect of different starting materials, synthesis conditions such as calcination time, temperature, and cooling rate on the final δ^18^O values of U_3_O_8_. The average δ^18^O values of U_3_O_8_ synthesized from UO_3_ and UNH are 8.94 ± 0.50‰ and 8.15 ± 0.66‰, respectively. The similarity of the δ^18^O values of U_3_O_8_ obtained for both preparation routes emphasizes that a common external source determines the oxygen isotopic composition. The similar δ^18^O values obtained for U_3_O_8_ synthesized from different isotopically spiked nitric acids imply that the final isotopic composition of U_3_O_8_ is independent of the starting material isotopic composition and further suggest the involvement of another oxygen source during calcination. Our kinetic experiments show that within 30 min, a stable δ^18^O value is achieved and remains stable over calcination times of 0.5 to 168 h and at calcination temperatures between 650 and 750 °C. It suggests that a fast oxygen isotope exchange occurs in this system. The effect of cooling profiles on the oxygen isotopic composition is determined by changing the cooling rate from 750 °C to room temperature, between 2.5 min to 33 h. Our results show that the cooling rate of U_3_O_8_ changes the final δ^18^O value by ∼30‰, suggesting that the cooling profile is the main factor governing the final δ^18^O value in U_3_O_8_ in the production process. The resetting of the oxygen isotope toward lighter compositions can be explained by the uranium mass effect. This study contributes to the development of a new signature to be used in nuclear forensic investigations.
